# CALENDAR

**DOI:** 10.1038/sj.bjc.6690703

**Published:** 1999-09


					12-15 December 1999
XV Asia Pacific Cancer Conference
Chennai (Madras), India
Further information from:
Secretary General, XV APCC Secretariat, Cancer Institute (WIA)
(Annex), 18 Sardar Patel Road, Chennai (Madras) 600-036, India.
Tel: + 91 44 2350131; Fax: + 91 44 4912085; Website:
http//apcc.uicc.org; E-mail: cancer_institute_wia@vsnl.com
13-15 December 1999
GENES & CANCER 99 - UK Molecular Biology &
Cancer Network Meeting XV1
University of Warwick, UK
Sessions include: Gene Expression* Checkpoints & DNA
Repair* Tumour Suppressor Genes* Beyond the Genome
Poster and Trade Exhibition
Poster Abstracts deadline: 22 October 1999;
Registration deadline: 3 November 1999
Further information from:
Dr Helen Hurst, ICRF, Hammersmith Hospital, Du Cane Road,
London W12 0NN, UK. Fax: + 44 (0) 181 383 3258; Website:
www.icr.ac/ukmbcn/info.htm
Errata
Br J Cancer 80(10): 1672-1677
Detection of regional melanoma metastases by ultrasound B-scan,
cytology or tyrosinase RT-PCR of fine-needle aspirates
C Voit, A Schoengen, M Schwurzer, L Weber, T Mayer and
TM Proebstle
The correct author addresses are listed below:
C Voit1
, A Schoengen1
, M Schwurzer1
, L Weber3
, T Mayer2
and
TM Proebstle4
1
Department of Dermatology of the Charite, Humboldt University
Berlin, Germany; 2
Department of Medical Oncology, Armed
Forces Hospital Ulm, Germany; 3
Department of Dermatology,
University of Ulm, Germany; 4
Department of Dermatology of
Mainz, Germany
Br J Cancer 80(1/2): 262-268
Psychological, clinical and pathological effects of relaxation
training and guided imagery during primary chemotherapy
LG Walker, MB Walker, K Ogston, SD Heys, AK Ah-See,
ID Miller, AW Hutcheon, TK Sarkar and O Eremin
The sentence in the Results section, under the heading
Psychological and psychiatric aspects, fourth paragraph, should
read:
There was a strikingly low level of abnormal scores after
chemotherapy for anxiety (5%) and depression (1%). Using a cut-
off score of 18 for combined scores of anxiety and depression to
identify clinically significant disorder (Hopwood et al, 1991), 4%
obtained an abnormal score before and 4% after chemotherapy.
This paper should also have an Acknowledgement section which
reads:
The authors are grateful to all the patients who participated in the
study and to the Cancer Research Campaign who provided
funding.
CALENDAR
376
British Journal of Cancer (1999) 81(2), 376
(c) 1999 Cancer Research Campaign
Article no. bjoc.1999.0703

				

